# Cerebrospinal Fluid Penetration and Combination Therapy of Entrectinib for Disseminated *ROS1/NTRK*-Fusion Positive Pediatric High-Grade Glioma

**DOI:** 10.3390/jpm10040290

**Published:** 2020-12-18

**Authors:** Lisa Mayr, Armin S. Guntner, Sibylle Madlener, Maria T. Schmook, Andreas Peyrl, Amedeo A. Azizi, Karin Dieckmann, Dominik Reisinger, Natalia M. Stepien, Kathrin Schramm, Anna Laemmerer, David T. W. Jones, Jonas Ecker, Felix Sahm, Till Milde, Kristian W. Pajtler, Mirjam Blattner-Johnson, Miroslav Strbac, Christian Dorfer, Thomas Czech, Dominik Kirchhofer, Lisa Gabler, Walter Berger, Christine Haberler, Leonhard Müllauer, Wolfgang Buchberger, Irene Slavc, Daniela Lötsch-Gojo, Johannes Gojo

**Affiliations:** 1 Department of Pediatrics and Adolescent Medicine and Comprehensive Center for Pediatrics, Medical University of Vienna, 1090 Vienna, Austria; lisa.mayr@meduniwien.ac.at (L.M.); sibylle.madlener@meduniwien.ac.at (S.M.); andreas.peyrl@meduniwien.ac.at (A.P.); amedeo.azizi@meduniwien.ac.at (A.A.A.); dominik.reisinger@meduniwien.ac.at (D.R.); natalia.stepien@meduniwien.ac.at (N.M.S.); anna.laemmerer@meduniwien.ac.at (A.L.); dominik.kirchhofer@meduniwien.ac.at (D.K.); irene.slavc@meduniwien.ac.at (I.S.); 2 Department of Medicine I, Institute of Cancer Research, Medical University of Vienna, 1090 Vienna, Austria; lisa.gabler@meduniwien.ac.at (L.G.); walter.berger@meduniwien.ac.at (W.B.); 3 Comprehensive Cancer Center-Central Nervous System Tumors Unit, Medical University of Vienna, 1090 Vienna, Austria; 4 Institute of Analytical Chemistry, Johannes Kepler University, 4020 Linz, Austria; armin_sebastian.guntner@jku.at (A.S.G.); wolfgang.buchberger@jku.at (W.B.); 5 Division of Neuroradiology and Musculoskeletal Radiology, Department of Biomedical Imaging and Image-Guided Therapy, Medical University of Vienna, 1090 Vienna, Austria; maria.schmook@meduniwien.ac.at; 6 Department of Radiotherapy, Medical University of Vienna, 1090 Vienna, Austria; karin.dieckmann@meduniwien.ac.at; 7 Hopp Children’s Cancer Center Heidelberg (KiTZ), 69120 Heidelberg, Germany; kathrin.schramm@kitz-heidelberg.de (K.S.); david.jones@dkfz.de (D.T.W.J.); till.milde@med.uni-heidelberg.de (T.M.); k.pajtler@kitz-heidelberg.de (K.W.P.); m.blattner-johnson@kitz-heidelberg.de (M.B.-J.); 8 Pediatric Glioma Research Group, German Cancer Research Center (DKFZ), 69120 Heidelberg, Germany; 9 Clinical Cooperation Unit Pediatric Oncology, Hopp Children’s Cancer Center Heidelberg (KiTZ), 69120 Heidelberg, Germany; j.ecker@dkfz.de; 10 Department of Neuropathology, Institute of Pathology, University Hospital Heidelberg, 69120 Heidelberg, Germany; Felix.Sahm@med.uni-heidelberg.de; 11 Clinical Cooperation Unit Neuropathology, German Consortium for Translational Cancer Research (DKTK), German Cancer Research Center (DKFZ), 69120 Heidelberg, Germany; 12 Division of Pediatric Neurooncology, German Cancer Research Center (DKFZ), 69120 Heidelberg, Germany; 13 Department of Pediatric Oncology, Hematology, and Immunology, University Hospital Heidelberg, 69120 Heidelberg, Germany; 14 Department of Laboratory Medicine and Pathology, Tree Top Hospital, Hulhumale 23000, Maldives; miroslavstrbac@yahoo.com; 15 Department of Neurosurgery, Medical University of Vienna, 1090 Vienna, Austria; christian.dorfer@meduniwien.ac.at (C.D.); thomas.czech@meduniwien.ac.at (T.C.); 16 Division of Neuropathology and Neurochemistry, Department of Neurology, Medical University of Vienna, 1090 Vienna, Austria; christine.haberler@meduniwien.ac.at; 17 Department of Pathology, Medical University of Vienna, 1090 Vienna, Austria; leonhard.muellauer@meduniwien.ac.at

**Keywords:** *NTRK* fusion, *ROS1* fusion, entrectinib, radiotherapy, CSF penetrance, targeted therapies, trametinib, abemaciclib

## Abstract

Targeting oncogenic fusion-genes in pediatric high-grade gliomas (pHGG) with entrectinib has emerged as a highly promising therapeutic approach. Despite ongoing clinical studies, to date, no reports on the treatment of cerebrospinal fluid (CSF) disseminated fusion-positive pHGG exist. Moreover, clinically important information of combination with other treatment modalities such as intrathecal therapy, radiotherapy and other targeted agents is missing. We report on our clinical experience of entrectinib therapy in two CSF disseminated *ROS1/NTRK*-fusion-positive pHGG cases. Combination of entrectinib with radiotherapy or intrathecal chemotherapy appears to be safe and has the potential to act synergistically with entrectinib treatment. In addition, we demonstrate CSF penetrance of entrectinib for the first time in patient samples suggesting target engagement even upon CSF dissemination. Moreover, in vitro analyses of two novel cell models derived from one case with *NTRK*-fusion revealed that combination therapy with either a MEK (trametinib) or a CDK4/6 (abemaciclib) inhibitor synergistically enhances entrectinib anticancer effects. In summary, our comprehensive study, including clinical experience, CSF penetrance and in vitro data on entrectinib therapy of *NTRK/ROS1*-fusion-positive pHGG, provides essential clinical and preclinical insights into the multimodal treatment of these highly aggressive tumors. Our data suggest that combined inhibition of *NTRK/ROS1* and other therapeutic vulnerabilities enhances the antitumor effect, which should be followed-up in further preclinical and clinical studies.

## 1. Introduction

Molecular profiling has significantly improved diagnosis and prognostic prediction of pediatric brain tumors and has therefore already been implemented in the current as well as the upcoming WHO classification of tumors of the central nervous system (CNS) [1]. Gene fusions, including the neurotrophic tyrosine kinase (*NTRK*) family or c-ros oncogene 1 (*ROS1*), are relatively rare, yet their therapeutic impact has been proven in multiple solid tumor types [2,3,4]. *NTRK* gene fusions occur with a prevalence of less than 1% across all tumor types [5,6]. With respect to brain tumors, the estimated prevalence of *NTRK*-fusions is 0.55 to 2% for gliomas and neuroepithelial tumors [5]. In the subgroup of infant hemispheric glioma (IHG), nearly two-thirds of cases harbor molecular alterations of anaplastic lymphoma kinase (*ALK*), *NTRK*, *ROS1* or tyrosine-protein kinase *MET* [7,8]. Adult and infant *NTRK*-fused gliomas are primarily located in the hemispheres with high-grade histology, whereas in older children, a more diverse anatomic distribution and low to high-grade histologic grades are found [9]. Adjuvant chemotherapy (CT) after surgery is the first approach in infants and allows delay or even avoidance of radiotherapy (RT) with a 5-year overall survival (OS) of 25.0% and 42.9% in *ROS1*- and *NTRK*-driven tumors, respectively [7,10,11]. In older pediatric patients, treatment consisting of surgery, RT and CT is applied, resulting in long-term survival rates around 10% [12]. Concomitant genomic alterations of *NTRK*-fused gliomas are more frequent in adult patients and high-grade tumors and include, among others, *CDKN2A/B* loss, *TERT* promoter mutation, *TP53* mutation/biallelic inactivation/loss, *PTEN* loss/mutation/biallelic inactivation, *EGFR* amplification, *ATRX* mutation, *RB1* loss, and *PIK3CA* mutation [9].

As *ROS1* or *NTRK* alterations are also major oncogenic drivers in other solid tumor types, targeted therapies have already been developed and evaluated within clinical studies. Larotrectinib and entrectinib, two highly specific TRK inhibitors, are FDA and EMA approved in patients with tumors harboring a *TRK* fusion or a *ROS1*-fusion in the case of entrectinib [13]. However, neoplasms in the brain are protected by the blood brain barrier (BBB), and the ability to pass this tissue layer is of utmost importance for targeted therapies [14]. Moreover, the penetrance of cerebrospinal fluid (CSF) may be particularly relevant for the treatment of leptomeningeal disseminated tumors. We have recently shown that CSF concentrations of small molecules can be reliably detected via samples obtained from an Ommaya reservoir, but data for CSF penetrance of *TRK* inhibitors in brain tumor patients is still lacking [15]. Entrectinib was designed to cross the BBB and displayed an objective response rate of 79% across different adult and pediatric solid tumors as well as efficacy in CNS tumors [16].

To date, no reports on the treatment of leptomeningeal dissemination in fusion-positive pediatric high-grade glioma (pHGG) exist. Moreover, clinically important information of potential combinations with other treatment modalities such as intrathecal therapy, RT and other targeted agents is missing. Here we report on our experience in treating two pediatric patients with leptomeningeal disseminated pHGG with entrectinib. Moreover, we demonstrate CSF penetration of entrectinib for the first time in a real-world setting in one patient and provide insights into resistance patterns and emerging therapeutic vulnerabilities upon entrectinib treatment of pediatric glioma.

## 2. Methods and Materials

### 2.1. Patient Samples and Characteristics

Both cases were treated at the Department of Pediatrics and Adolescent Medicine of the Medical University of Vienna (MUV) (one was referred at progression for entrectinib treatment after previous treatment at other centers). Clinical information was obtained from patient charts. The extent of surgical resection was defined on postoperative magnetic resonance imaging (MRI) performed within 48 h as gross total resection (GTR, no obvious residual tumor), partial resection (PR, 10–50% residual tumor) and biopsy (>50% residual tumor). The study was approved by the local institutional review board of the Medical University of Vienna (EK Nr. 1244/2016). Informed consent was obtained for all patients and/or legal representatives.

### 2.2. Histopathology

The histopathological diagnoses were assessed by experienced neuropathologists according to the 2016 WHO classification. For diagnostic purposes, a routine histopathological examination on formalin-fixed paraffin-embedded (FFPE) tissue was performed, including immunohistochemical (IHC) analysis with vimentin, GFAP, S100, EMA, CKAE1/3, Desmin, Olig2, MAP2, CD31, CD34, CD99, p53, bcl-2, NCAM, IDH1, BCAT1, ATRX, BCOR, STAT6, BAF47 (INI1), SMARCA4 (BRG1), EGFR, PD-L1.

### 2.3. Next-Generation Sequencing (NGS)

In case 2, NGS was performed on the tissue of the left frontal metastasis with oncomine comprehensive assay v3 (Thermo Fisher Scientific, Waltham, MA, USA) according to the manufacturer’s instructions [17]. DNA and RNA extracted from FFPE tissue were used. Oncomine comprehensive assay v3 detects single nucleotide variants (SNV), copy number variations (CNV), gene fusions, and indels from 161 unique cancer-associated genes [17].

FoundationOne^®^ CDx (F1CDx, Foundation Medicine, Inc., Cambridge, UK) analysis was conducted with the biopsy of the left fronto-median metastasis of case 2. F1CDx is an NGS-based in vitro diagnostic device for detection of substitutions, insertion and deletion alterations (indels), and copy number alterations (CNAs) in 324 genes and select gene rearrangements, as well as genomic signatures including microsatellite instability (MSI) and tumor mutational burden (TMB) using DNA isolated from FFPE tumor tissue specimens [18]. Moreover, the left fronto-median metastasis of case 2 was analyzed at the Deutsches Krebsforschungszentrum (DKFZ) through the individualized therapy for relapsed Malignancies in childhood (INFORM) registry [19]. The first relapse of case 1 was analyzed via the Pediatric Targeted Therapy 2.0 (PTT 2.0) registry study (NCT-2016-041 4) at the DKFZ.

### 2.4. High-Performance Liquid Chromatography-Mass Spectrometry (HPLC-MS) Analysis

The quantitation of entrectinib was performed with a modified version of the general assay described earlier by our group [15]. First, CSF specimens were treated with a five-fold surplus of methanol. After homogenization and centrifugation, 7 µL of supernatants were submitted to HPLC-MS analysis using a 1260 Infinity II HPLC (Agilent Technologies, Santa Clara, CA, USA) hyphenated to a 6460 triple quadrupole mass spectrometer (Agilent Technologies). For chromatography, a Poroshell 120 column (EC-C18, 2.7 µm, 3 × 150 mm, Agilent Technologies) in combination with a gradient system of acetonitrile and water (both modified with 0.1% formic acid) was used. Sensitivity, as well as specificity of the assay, were assured in method development. Possible matrix effects were compensated with matrix-matched calibration.

### 2.5. Cell Models

Tumor tissues for analyses and establishment of patient-derived cell models were derived from patients treated at the MUV. The primary gliosarcoma cell lines originating from case 2 VBT247 (primary tumor at diagnosis) and VBT363 (3rd recurrence under treatment with entrectinib) were cultured in RPMI-1640 medium (Sigma-Aldrich, St. Louis, MO, USA) supplemented with 10% fetal calf serum (FCS, Gibco, Thermo Fisher Scientific) at 37 °C in a 5% CO_2_ incubator. The cell models were regularly checked for mycoplasma contamination.

### 2.6. ATP Assay

Cells were plated in triplicates (4 × 10^4^ cells/mL) in 100 μL growth medium per well in 96-well plates and allowed to attach for 24 h. All drugs were purchased from Selleck Chemicals (Houston, TX, USA). Entrectinib (0 to 10 µM), trametinib (0 to 10 μM), everolimus (0 to 50 µM) and abemaciclib (0 to 10 µM) were added alone or in different combination regimens in 100 μL growth medium with 10% FCS and cells were exposed for 72 h. The proportion of viable cells was determined by ATP assay following the manufacturer’s recommendations (“CellTiter-Glo^®^ luminescent cell viability assay”, Promega, Madison, WI, USA). Luminescence was measured at 1000 nm at the Tecan infinite 200Pro (Zurich, Switzerland). Raw data were analyzed using GraphPad Prism 8.0 software (GraphPad Software Inc., La Jolla, CA, USA). Results are given as mean +/− SD and were normalized to untreated control cells. Cytotoxicity was expressed as IC_50_-values calculated from full dose–response curves (drug concentrations inducing a 50% reduction of the cell number in comparison to the untreated control cells). The interaction between the activities of combined drugs is expressed by the combination index (CI) as published by Chou [20] using CalcuSyn software (Biosoft, Ferguson, MO, USA). CI < 0.9, CI = 0.9–1.2 or CI > 1.2 represent synergism, additive effects and antagonism, respectively.

### 2.7. Colony Formation Assay

A low-density of cells (1 × 10^4^ cells per well) was seeded in 500 μL growth medium in triplicates in 24-well plates. Following 24 h of recovery, 1 µM entrectinib, 1 μM trametinib, 1 µM abemaciclib, 5 μM everolimus or different combination treatments were applied and repeated after 7 days incubation time. On day 14 of exposure, cells were washed twice with 1×PBS, fixed with methanol at 4 °C and stained with crystal violet (10 μg crystal violet per 10 mL PBS). Photomicrographs were taken using a Nikon D3200 camera, and densitometric quantification of the images was analyzed with ImageJ software (Image J2, Wayne Rasband, NIH, Bethesda, MD, USA).

### 2.8. Statistical Analysis

Statistical analysis was performed using GraphPad Prism 8.0. All experiments were carried out independently at least three times. All data are expressed as mean +/− S.D. Statistical significance of differences was analyzed by using one-way ANOVA. A *p*-value < 0.05 was considered statistically significant. Throughout the study the following classification is used: *, *p* < 0.05; **, *p* < 0.01 ***, *p* < 0.001, ****, *p* < 0.0001.

## 3. Results

### 3.1. Clinical Characteristics and Response to Entrectinib

**Figure 1:**
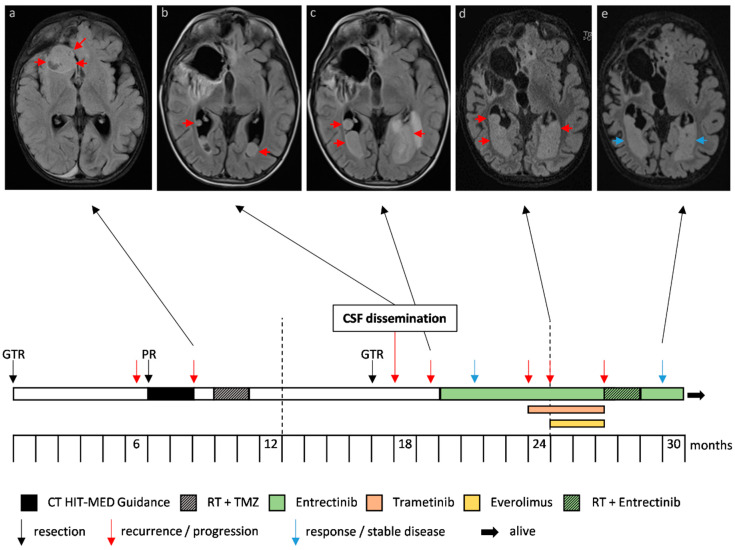
Case 1, an infantile hemispheric glioma harboring a *ROS1:ARCN1* fusion. Progression and therapy response of metastases in the lateral ventricles depicted in axial T2 weighted magnetic resonance images with cerebrospinal fluid (CSF) suppression (**a**–**e**). The arrows indicate tumor (red, progression; blue, response/stable disease). The timeline indicates the different treatment strategies and interventions. The frontobasal metastasis (**a**) was resected.

Case 1 (overview in Figure 1, including H&E and p53 IHC staining in Figure S1A,B), diagnosed with an IHG at two years of age, received a GTR and consecutive follow-up as first-line therapy. Due to a local recurrence detected six months after diagnosis, the patient was transferred to the Heidelberg University Hospital and was treated with PR and CT according to the HIT-MED Guidance protocol (ClinicalTrials.gov NCT02417324). Following two courses of CT, tumor progression was observed, and therapy was switched to local proton irradiation (54 Gy to the tumor bed) and concomitant oral temozolomide (TMZ) (75 mg/m^2^). Molecular analysis of tumor tissue revealed a *ROS1:ARCN1* fusion, and therapy with a ROS1-inhibitor was therefore suggested but not feasible due to the unavailability of matching clinical studies in Europe at this time point. Consequently, the patient was referred back to the external center. Six months later, the patient developed focal seizures prompting a GTR of the residual tumor. Histopathological examination revealed 15% vital tumor cells. Four weeks after re-operation, the cranial follow-up MRI revealed extensive CSF dissemination in both lateral ventricles (Figure 1). At this time, the patient was referred to the Medical University of Vienna for treatment with entrectinib and received a follow-up MRI of the craniospinal axis only six weeks later, just before his first appointment in Vienna. Within this limited timeframe, the tumor had rapidly progressed, now showing progressive CSF dissemination in both lateral ventricles, the fourth ventricle, and the spinal dural sac. Treatment with entrectinib (400 mg daily) was immediately initiated and well-tolerated. Subsequently, tumor growth was drastically reduced and showed partial response. However, regular surveillance MRIs revealed slow tumor progression over the following months. Due to the previous detection of mitogen-activated kinase (MAPK) pathway activation in comprehensive molecular profiling and an inactivating *PTEN* mutation as well as the loss of chromosome 10, therapy was augmented with trametinib and everolimus. The therapeutic approach with entrectinib, trametinib and everolimus resulted in an almost stable disease. Unfortunately, the patient developed a massive drug rash and therapy with trametinib and everolimus had to be terminated after 14 weeks of trametinib and 10 weeks of everolimus. Subsequently, the patient was treated with local RT (whole ventricular dose 36 Gy and 42 Gy focal to the metastasis in the dural sac) and concomitant entrectinib. Eight months after RT, the patient currently shows stable disease with entrectinib monotherapy and remains in good clinical condition.

**Figure 2:**
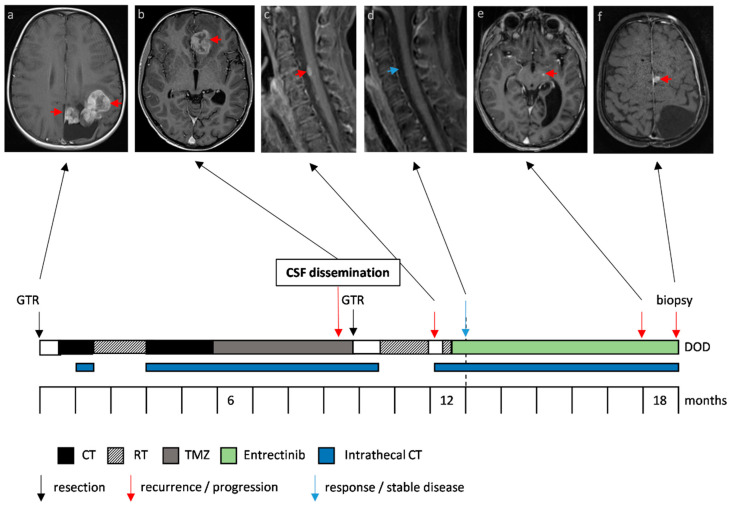
Case 2, a gliosarcoma harboring an *EML4:NTRK3* fusion. Therapy response and progression of metastases under treatment with entrectinib is depicted in axial T1 weighted contrast-enhanced magnetic resonance images of the brain and sagittal images of the cervical spine. The red arrows indicate the primary tumor (**a**) and metastases (**b**,**c**,**e**,**f**), the blue arrow the regression of the perimedullary metastasis (**d**). The timeline indicates the different treatment strategies and interventions.

Case 2 (overview depicted in Figure 2, including H&E and p53 IHC staining in Figure S2A,B), presented at the age of nine years with a left parieto-occipital solid cystic tumor, diagnosed as gliosarcoma. GTR was followed by two cycles of ICE (ifosfamide, carboplatin, etoposide) [21] and focal RT (59.4 Gy to the tumor bed) as first-line therapy. Adjuvant CT was changed to TMZ (150–200 mg/m^2^ on 5 consecutive days every 28 days) because of prolonged aplasia and was augmented with intraventricular therapy via an Ommaya reservoir consisting of etoposide, aqueous cytarabine and topotecan every other week [22,23,24,25]. Ten months after the initial diagnosis, metastasis in the left frontal lobe occurred, and treatment consisting of GTR followed by focal RT (59.4 Gy to the tumor bed) was applied. Intrathecal therapy was discontinued during RT. However, two weeks following re-irradiation, the patient reported massive, constant and irrepressible pain in his right leg. MRI revealed distinct cerebral and spinal leptomeningeal dissemination with the biggest metastasis located at the level of thoracic vertebra 9 (Th9), causing compression of the spinal cord. Again, focal RT was applied (five times 5 Gy) only to the symptomatic lesion at level Th9. Further molecular analysis of the tumor tissue revealed an *EML4:NTRK3* fusion. Treatment was switched to entrectinib (600 mg daily) augmented with intrathecal therapy every other week. No side effects were observed, and the clinical condition substantially improved after RT and upon treatment with entrectinib making it even possible for the patient to exert sports only eight weeks after RT and initiation of entrectinib. Neuroimaging revealed a regression of the cerebral and spinal leptomeningeal deposits (Figure 2 depicts the response in the cervical region that had not been irradiated). Five months after the initiation of entrectinib, the patient developed ataxia and dizziness. Further leptomeningeal dissemination with new lesions in the left medial superior frontal gyrus, right postcentral, bilateral cerebellar and basal brainstem regions was observed (Figure 2). In order to detect further potential therapeutic targets, biopsy and subsequent molecular analysis of the left fronto-medial metastasis was performed. Unfortunately, the patient succumbed to his disease due to the rapid tumor progression.

### 3.2. Molecular Diagnostics and Next Generation Sequencing

With respect to case 1, NGS within the PTT2.0 registry study (NCT-2016–0414) revealed a *ROS1:ARCN1* fusion in the IHG. The tumor was negative for *IDH1*(*R132*) mutation and *MGMT* unmethylated. Furthermore, a hot spot mutation in the *PTEN* gene and a heterozygous loss (LOH) of chromosome 10, causing a combined loss of *PTEN* function in the tumor was detected. Immunohistochemical analyses showed an aberrant activation of the MAPK pathway. Table 1 shows an overview of histopathology, molecular characteristics and next-generation sequencing of our patients. The respective copy number plot derived from methylation array analysis is depicted in Figure S3.

In case 2, Oncomine Comprehensive v3 analysis of the local left frontal gliosarcoma recurrence detected an *EML4:NTRK3* fusion, a *CDKN2A/B* loss and an *MRE11A* alteration of unknown significance. Analysis of the left fronto-median metastasis that developed under treatment with entrectinib via the INFORM registry at the DKFZ revealed the already known *EML4:NTRK3* fusion without resistance mutation, the homozygous *CDKN2A*/*B* deletion already detected in the primary tumor, an *INSR* mutation (p. D601Y, VAF = 0.4), an *NF2* splicing mutation (c.522 + 1G > C, VAF = 0.66, resulting in exon-skipping), and *AURKC*, *IGF1* and *TGFB3* mRNA overexpression. Analysis of the same biopsy via F1CDx confirmed the homozygous *CDKN2A/B* loss and the *NF2* mutation. The respective copy number plot derived from methylation array analysis is depicted in Figure S4.

### 3.3. CSF Penetration of Entrectinib

**Figure 3:**
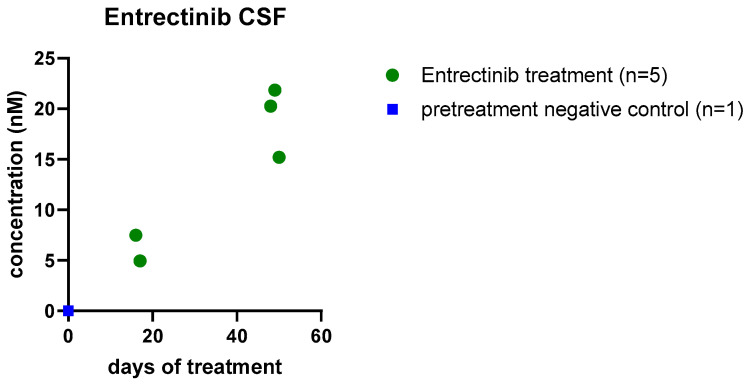
CSF penetrance of entrectinib. Entrectinib concentration (nM) detected in the cerebrospinal fluid of case 2 by high-performance liquid chromatography-mass spectrometry (HPLC-MS) as previously described [15]. Entrectinib levels increase over time of intake. Individual points depict entrectinib concentrations in independent samples obtained via an Ommaya reservoir before administration of intrathecal therapy.

As already mentioned, penetrance of the brain is of essential importance in the treatment of CNS tumors, but so far, evidence for CSF penetration of entrectinib has been limited to preclinical studies [26]. Using our previously published approach [15], we analyzed entrectinib CSF penetration in 5 samples of case 2 obtained before administration of intrathecal therapy. We found entrectinib concentrations in the low nM range, which increased during ongoing therapy (Figure 3). As case 1 has no Ommaya reservoir and underwent no surgery at the MUV, CSF samples were not available of this patient for HPLC-MS analysis.

### 3.4. Impact of Entrectinib and Combination with Targeted Therapies on NTRK-Fusion Positive pHGG Cell Viability and Proliferation

**Figure 4:**
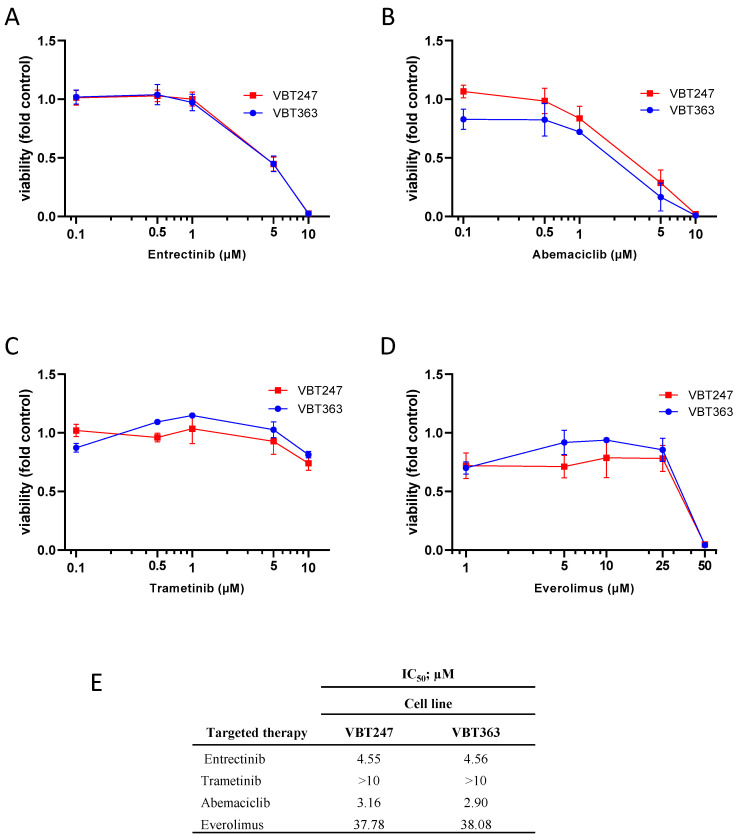
Effect of single-agent targeted therapies in *EML4:NTRK3* positive cell lines. The impact on cell viability of (**A**) entrectinib, (**B**) abemaciclib, (**C**) trametinib and (**D**) everolimus on the primary tumor (VBT247) and the 3rd recurrence under treatment with entrectinib (VBT363) was tested after 72 h incubation by ATP assay in triplicates. (**E**). The inhibitory effect is expressed as IC_50_ values calculated from full dose–response curves (drug concentrations inducing a 50% reduction of the cell number in comparison to the untreated control cells).

Under standard culture conditions, treatment with increasing concentrations of entrectinib for 72 h caused a dose-dependent decrease of cell viability in both gliosarcoma cell lines that were derived from two consecutive surgeries of case 2, VBT247 (primary diagnosis) and the 3rd recurrence under treatment with entrectinib (VBT363); growth curves and IC_50_ values are depicted in Figure 4A,E. Interestingly, the antiproliferative effect of entrectinib showed no difference in the tumor cell model VBT247 derived from the primary tumor as compared to VBT363, the cell model established following clinical resistance to entrectinib. As both cell lines harbor a homozygous loss of *CDKN2A/B* we tested the effect of the CDK4/6 inhibitor abemaciclib on cell viability (Figure 4B,E). VBT247 and VBT363 showed a distinct sensitivity against CDK4/6 inhibition. In contrast, both gliosarcoma cell models were comparably insensitive to treatment with trametinib and everolimus with IC_50_ values above 10 µM clearly exceeding clinically achievable doses in the short-term exposure as depicted in Figure 4C–E.

**Figure 5:**
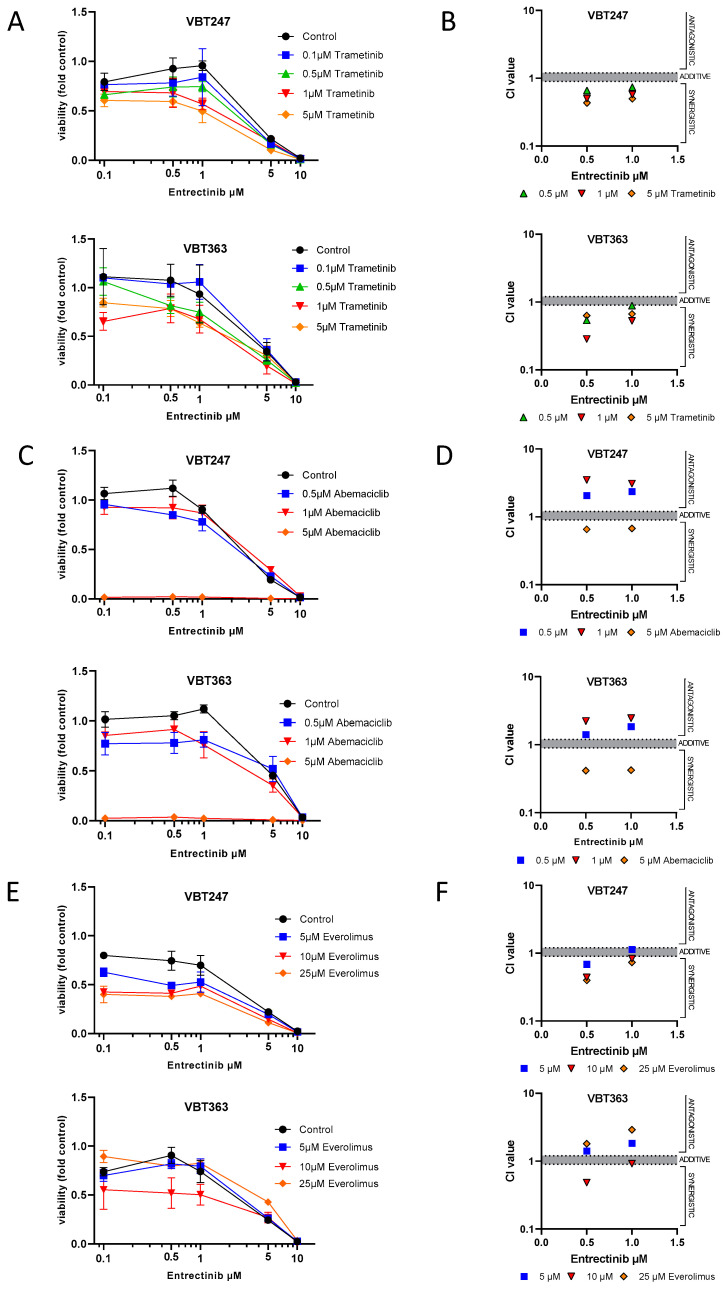
Effect of entrectinib and combination treatment on cell viability and toxicity. Impact of entrectinib treatment in combination with (**A**) trametinib, (**C**) abemaciclib, (**E**) everolimus on cell viability in triplicates in the indicated cell models measured by ATP-based survival assays upon 72 h drug exposure. Combination index (CI) based on the distinct drug combinations (**B**) trametinib, (**D**) abemaciclib, (**F**) everolimus were calculated as published [20]. CI values < 0.9 indicates synergistic, CI = 0.9–1.2 additive and CI > 1.2 antagonistic effects.

The impact of a combined application of the NTRK inhibitor entrectinib with other targeted therapies (e.g., abemaciclib, trametinib and everolimus) was tested for short- (72 h) and long-term exposure (14 days) by ATP and clonogenicity assays, respectively. NTRK inhibition distinctly synergized with trametinib in VBT247 and VBT363 cells (growth curves and combination indices shown in Figure 5A,B). In contrast, entrectinib generally antagonized the effect of abemaciclib in low concentrations but indeed synergized with abemaciclib in higher concentrations (5 µM abemaciclib) depicted in Figure 5C,D. Last, entrectinib showed additive to synergistic effects when combined with everolimus in VBT247 and VBT363 cells (Figure 5E,F).

**Figure 6:**
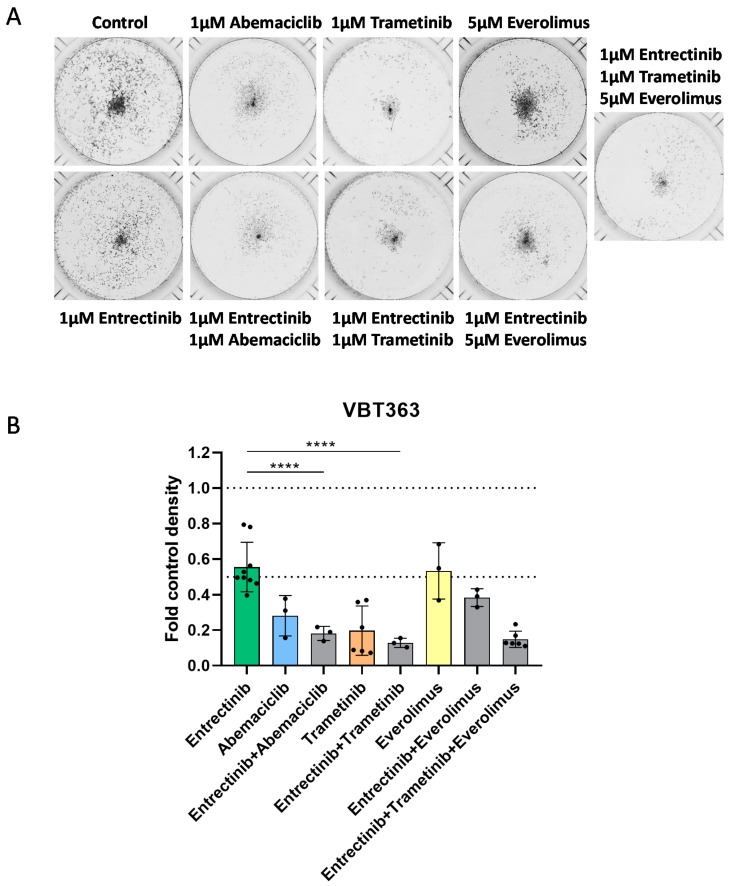
Effect of entrectinib and combination treatment on cell viability and toxicity. Effects of combined long-term application of entrectinib with trametinib, abemaciclib and/or everolimus in VBT363 cells were tested by clonogenicity assays in triplicates. (**A**) Cells were fixed and stained with crystal violet, and wells were photographed. (**B**) Densitometric quantification of photomicrographs shown in (**A**) using ImageJ2 software was assessed. Statistical significance of differences was analyzed by one-way ANOVA, ****, *p* < 0.0001.

In order to test antiproliferative effects in more detail, we performed a long-term exposure clonogenicity assay. Inhibitory effects were already detectable at 1 µM entrectinib and 1 µM abemaciclib in both gliosarcoma cell lines as depicted in Figure 6A,B. Furthermore, the sensitizing effect of trametinib and everolimus was distinctly increased in the clonogenicity assay and was already detectable at 1 µM trametinib and 5 µM everolimus (Figure 6A,B). The strong effect of trametinib alone was not further increased when combined with entrectinib.

## 4. Discussion

Targeting fusion-positive pHGG with small molecule inhibitors has emerged as a highly promising therapeutic approach to combat these aggressive and therapy refractory cancer types.

Herein, we report on two patients with leptomeningeal disseminated pHGG treated with entrectinib. Entrectinib, a selective pan TRK inhibitor, has already demonstrated significant responses in *NTRK*-fused tumors, including primary CNS tumors and CNS metastases [16,27,28,29]. The STARTRK-NG trial included four pHGG patients treated with entrectinib. All patients showed a radiographic response, including one complete response (2019, ASCO Annual Meeting Abstract #: 10009). To date, only a few reports describing primary *NTRK*-fused CNS tumors treated with either larotrectinib or entrectinib are available in the literature [8,30,31,32]. An adult patient with a *BCAN:NTRK1* fused glioneuronal tumor developed disease progression after eleven months of entrectinib [30]. Two reports describe patients suffering from a low-grade glioma (LGG), one with a *NACC2:NTRK* fusion showing more than 50% reduction in tumor volume and an *ETV6:NTRK3*-fused tumor with complete remission upon treatment with larotrectinib [8,31]. Moreover, in two *ETV6:NTRK3* fusion-positive pHGG, larotrectinib was administered. One patient experienced more than 70% tumor volume reduction and one disease progression [8,32]. In the pooled analysis of the STARTRK-2, STARTRK-1 and ALKA-371-001 trials, overall median progression-free survival in patients with CNS disease was 7.7 months, with most observed responses within the first or second treatment cycle [16]. This is in line with our observations showing the response of tumors with leptomeningeal dissemination after four weeks of entrectinib. The progression-free survival on entrectinib treatment observed in our gliosarcoma patient was 5 months, which is comparable to the observation in various trials [16]. In contrast, the tumor progression was markedly reduced but not totally blocked in our IHG patient, and treatment is currently ongoing 16 months after the start of entrectinib.

To explore the potential of entrectinib combination therapy in fusion-positive pHGG, we used two cell models derived from our case with *NTRK*-fusion. The observed in vitro sensitivity towards entrectinib was comparable to a report investigating an *ETV6:NTRK3* fusion-positive model [8]. Importantly, one model was derived from the primary tumor (VBT247), whereas the second model (VBT363) was derived from the progressive tumor during entrectinib treatment. Interestingly, both models demonstrated sensitivity towards entrectinib in vitro, suggesting that oncogenic *NTRK*-activation is still present in progressive tumors. Moreover, in a previous study investigating *BRAF*(*V600E*)-mutated high-grade glioma, we found a similar effect in comparing two models derived before and after treatment with a combination of dabrafenib and trametinib [33]. However, further studies, including long-term entrectinib exposure of these cell models, would be useful to more deeply dissect the resistance mechanisms of pHGG towards entrectinib.

Acquired resistance mutations to entrectinib in the kinase domain of the *NTRK* gene have been described in other tumor types [34,35,36]. Selitrectinib (LOXO-195) was designed to target these resistance mutations as well as the wild-type protein [35]. A pooled analysis revealed that all patients who developed resistance to larotrectinib had secondary *NTRK* mutations [37]. However, in our gliosarcoma patient with disease progression under entrectinib, no secondary *NTRK* mutation was detected, but molecular profiling revealed activation of alternative oncogenic pathways, including *NF2* and insulin receptor (*INSR*). Whether these effects are acquired events or whether resistant tumor cells emerge from primary subclones being intrinsically resistant—as, for example, cancer stem cells—which facilitated disease progression remains to be clarified [38,39,40]. The latter could indeed be present as we have already demonstrated high intratumoral heterogeneity within other pHGG types [38,39,40,41].

MAPK pathway activation by signal transducers not related to *NTRK* has already been described in other tumor types [42]. In the *ROS1:ARCN1* fusion patient from our study, a hot spot mutation in the *PTEN* gene and a heterozygous loss of chromosome 10 causing a loss of *PTEN* function, a frequent oncogenic event resulting in activation of the mammalian target of rapamycin (mTOR) pathway, as well as aberrant activation of the MAPK pathway were detected. Entrectinib monotherapy led to a massive deceleration of tumor growth, but stable disease was not achieved. Therefore, we treated our patient with a combined approach consisting of entrectinib, trametinib and everolimus. This combination was able to further reduce tumor growth and resulted in almost stable disease, but the combination was not well-tolerated as has been described for MEK inhibitors and mTOR inhibitors in an adult clinical trial [43] and had to be discontinued. Intriguingly, in our gliosarcoma cell model, entrectinib synergized with trametinib in the short-term exposure and distinctly decreased cell viability. In contrast, the effect of trametinib alone in the short-term exposure experiment showed IC_50_ values exceeding 10 µM. Interestingly, in the MAPK pathway-activated pLGG IC_50_ values for trametinib are in the low nm range suggesting activation of alternative pathways such as mTOR in fusion-positive pHGG [44]. Moreover, in the long-term exposure, already 1 µM of trametinib led to a distinct decrease in cell viability and markedly exceeded the effect of entrectinib alone. This points to a cytostatic effect of trametinib, rather than inducing apoptosis, as recently described for the treatment of glioblastoma cells [45]. Treatment with everolimus showed only limited effects on cell viability in the short- and long-term exposure experiments in our gliosarcoma cell models. In summary, our first preclinical studies on entrectinib combination treatment in pHGG suggest that a combined application of entrectinib and trametinib could be a promising treatment option for patients in the clinic. This effect is well described for the treatment of *BRAF*(*V600E*) mutated melanoma, and combination therapy of BRAF inhibitors and MEK inhibitors has become the standard of care as it is superior to monotherapy [46,47,48]. Based on the detected *CDKN2A/B* deletion, we further tested the potential of the CDK4/6 inhibitor abemaciclib, which showed strong efficacy against both gliosarcoma cell models and entrectinib distinctly synergized with 5 µM abemaciclib in vitro. Consequently, the combination therapy of CDK4/6 inhibitors could be a future strategy to overcome therapy resistance to NTRK inhibitors.

With respect to patient treatment, entrectinib has been shown to be generally well-tolerated, and the most common reported adverse events were dysgeusia, fatigue, constipation, diarrhea, dizziness, peripheral edema, weight gain and anemia [16]. In our cases, both patients had no entrectinib-related side effects. Although therapy with entrectinib and trametinib caused cutaneous side effects in our *ROS1:ARCN1* fusion patient, therapy could be continued with local cutaneous therapy. However, the addition of everolimus to the combination of entrectinib and trametinib markedly increased the cutaneous side effects and therapy with trametinib and everolimus had to be terminated. Dose interruption was necessary for almost 50%, and study discontinuation in 10% of patients treated with everolimus and trametinib due to cutaneous adverse events [49]. Therefore, the effect of entrectinib on the cutaneous side effects is unclear since they might have been caused by trametinib and everolimus alone.

To date, there are no reports on the combined administration of entrectinib and radiotherapy. The recommendation is to start entrectinib two weeks after radiotherapy. However, the fast progression of tumors and leptomeningeal spread in our two cases underline that an interruption of therapy may result in serious harm for patients suffering from these aggressive tumors. Hence, one patient continued treatment with entrectinib during the whole radiotherapy course, and we have not seen any side effects by the time of this report. The other patient started immediately after completing radiotherapy, also demonstrating no side effects. Consequently, in our experience combination of entrectinib and radiotherapy may be justified upon clinical need in these tumor types, which harbor a potential for rapid tumor growth.

Intratumoral target engagement remains one major obstacle for effective treatment of brain tumors, which are considered to be protected from anticancer drugs by the BBB. Entrectinib demonstrated CNS penetration capacity in preclinical models with repeated oral daily dosing [26,50]. In our study, we could demonstrate CSF penetrance of entrectinib in a patient for the first time. Entrectinib was detectable in the CSF with increasing concentrations over time in our gliosarcoma patient. The detected entrectinib concentrations were in the same range and even slightly higher, as reported in animal models [26]. Therefore, entrectinib appears to harbor potential in the therapy of *NTRK*-fused high-grade gliomas, particularly upon leptomeningeal dissemination. We could demonstrate that entrectinib is able to cross the BBB and reaches therapeutic doses for antitumor activity in the CSF. Moreover, our experience suggests that a combination of intrathecal therapy may be beneficial in cases with leptomeningeal dissemination. However, due to the limited number of cases described in this work, there are certain limitations to our observations. Further in-depth investigation and prospective clinical studies are necessary to further elucidate the role of entrectinib as well as possible combination therapies in *ROS1/NTRK*-fusion-positive pHGG.

## 5. Conclusions

Our comprehensive study, including clinical experience and in vitro data on therapy of *NTRK/ROS1*-fusion-positive pediatric high-grade gliomas, provides first insights into potential effective combination therapies. We show that combination with either trametinib or abemaciclib enhances the anticancer effects of entrectinib in vitro. Furthermore, a combination of entrectinib with radiotherapy or intrathecal chemotherapy is safe and appears to enhance the antitumor activity of entrectinib in cases with leptomeningeal dissemination.

## Figures and Tables

**Table 1:** Histopathologic, molecular characteristics and next-generation sequencing.

Case	Disease Status	Location	Histology	Gene-Fusion	Mutations	MGMT	Chromosomal Deletions	Molecular Findings	Cell Line
1	1st recurrence	right hemisphere	IHG	*ROS/ARCN1*	*PTEN*	unmeth.	10	MAPK-activation	-
2	primary tumor	left occipital	gliosarcoma	*EML4-NTRK3*	*MRE11A* (VUS)	unmeth.	*CDKN2A/B*	-	VBT247
2	3rd recurrence	left fronto-median	gliosarcoma	*EML4-NTRK3*	*INSR*, *NF2*	unmeth.	5 p, 8 p, 20, 22, *CDKN2A/B*	*AURKC*, *IGF1*, *TGFB3* overexpression	VBT363

## Supplementary Materials

The following are available online at https://www.mdpi.com/2075-4426/10/4/290/s1, Figure S1: H&E and p53 IHC staining of the *ROS1:ARCN1* fusion-positive infantile hemispheric glioma (200x), Figure S2: H&E and p53 IHC staining of the *EML4:NTRK3* fusion-positive gliosarcoma (200×), Figure S3: CNV-plot in the *ROS1:ARCN1* fusion-positive infantile hemispheric glioma as determined by methylation array of tumor tissue, Figure S4: CNV-plot in the *EML4:NTRK3* fusion-positive gliosarcoma as determined by methylation array of tumor tissue
