# Supplementary material for: Cerebrospinal Fluid Penetration and Combination Therapy of Entrectinib for Disseminated ROS1/NTRK-Fusion Positive Pediatric High-Grade Glioma
Source: J Pers Med. 2020 Dec 18;10(4):290. doi: 10.3390/jpm10040290 (PMC7766483; doi:10.3390/jpm10040290)
Supplement: Supplementary file 1 [file jpm-10-00290-s001.zip › jpm-1025097-supplementary.pptx]

## Slide 1
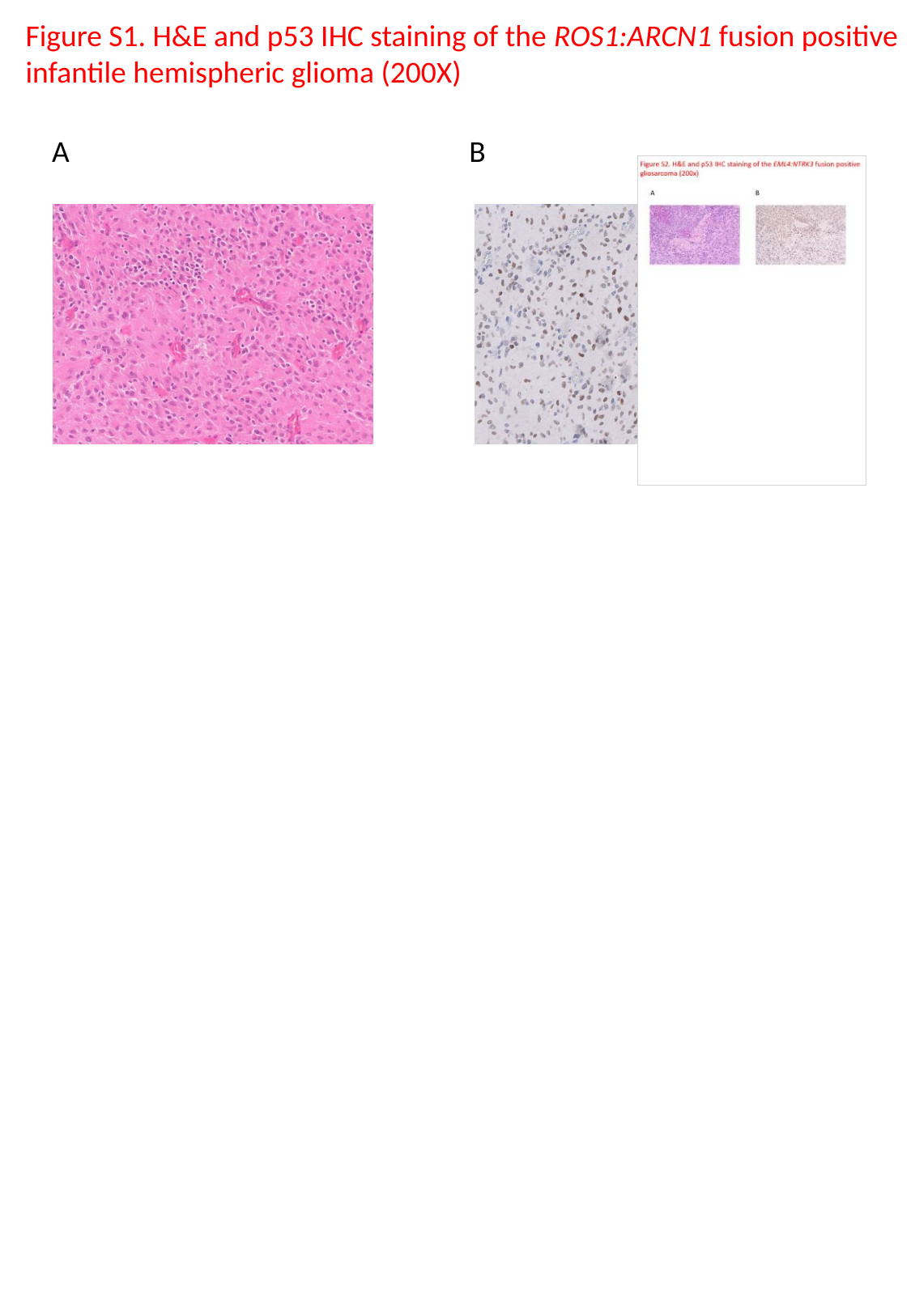

Figure S1. H&E and p53 IHC staining of the ROS1:ARCN1 fusion positive
infantile hemispheric glioma (200X)
A
B

## Slide 2
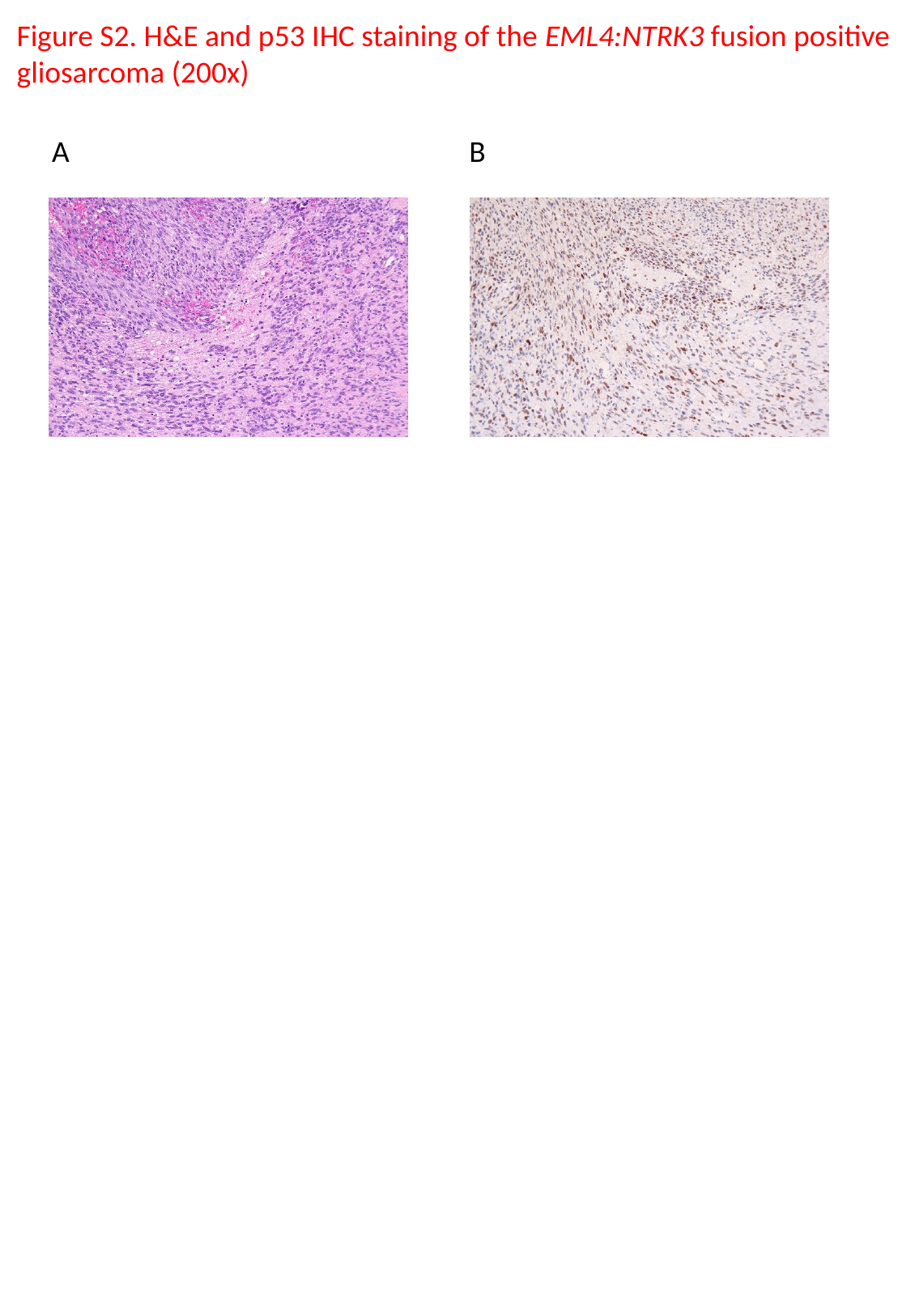

Figure S2. H&E and p53 IHC staining of the EML4:NTRK3 fusion positive
gliosarcoma (200x)
A
B

## Slide 3
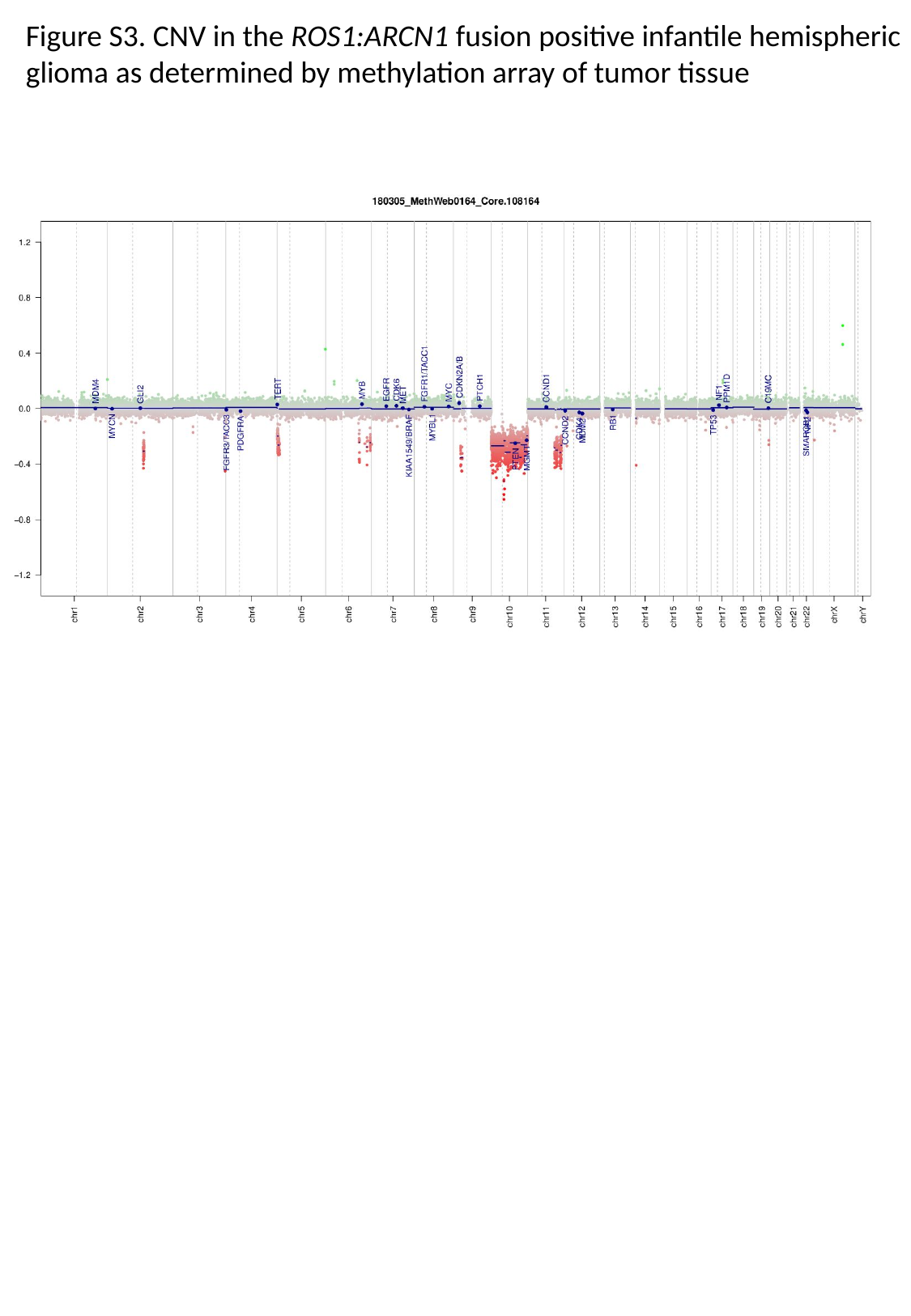

Figure S3. CNV in the ROS1:ARCN1 fusion positive infantile hemispheric
glioma as determined by methylation array of tumor tissue

## Slide 4
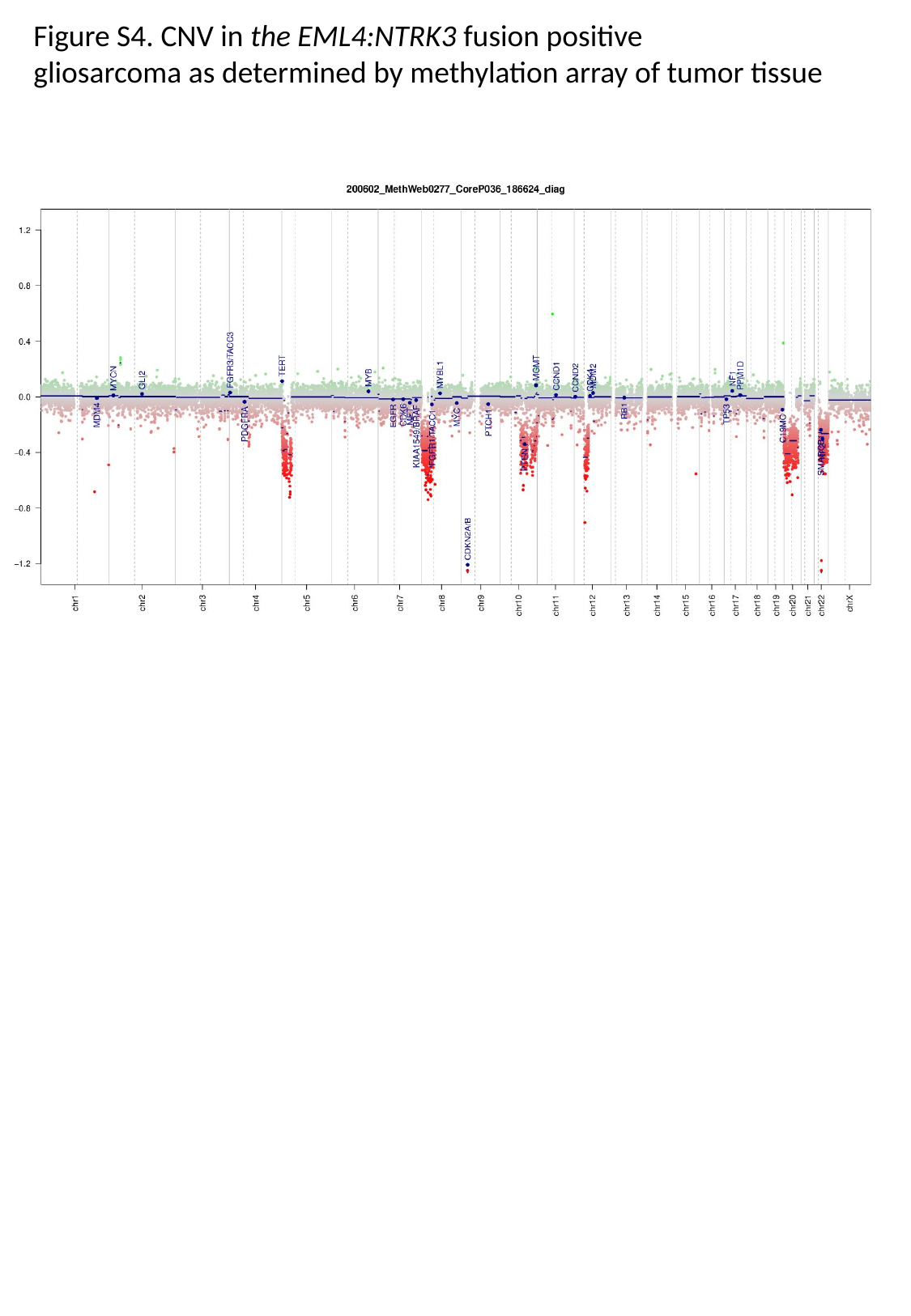

Figure S4. CNV in the EML4:NTRK3 fusion positive
gliosarcoma as determined by methylation array of tumor tissue
